# Effect of storage, temperature, and extraction kit on the phylogenetic composition detected in the human milk microbiota

**DOI:** 10.1002/mbo3.1127

**Published:** 2020-12-29

**Authors:** Katriona E. Lyons, Fiona Fouhy, Carol‐Anne O’ Shea, C. Anthony Ryan, Eugene M. Dempsey, R. Paul Ross, Catherine Stanton

**Affiliations:** ^1^ Teagasc Food Research Centre Moorepark, Fermoy, Co. Cork Ireland; ^2^ School of Microbiology University College Cork Cork Ireland; ^3^ APC Microbiome Ireland University College Cork Cork Ireland; ^4^ Department of Neonatology Cork University Maternity Hospital Cork Ireland

**Keywords:** extraction kit, human milk, microbiome, storage, temperature

## Abstract

Human milk is considered the optimum feeding regime for newborns and is a source of bacteria for the developing infant gastrointestinal tract. However, as with all low biomass samples, standardization across variabilities such as sample collection, storage, and extraction methods is needed to eliminate discrepancies in microbial composition across studies. The aim of this study was to investigate how different storage methods, temperatures, preservatives, and extraction kits influence the human milk microbiome, compared to fresh samples. Breast milk samples were processed via six different methods: fresh (Method 1), frozen at −80°C (Method 2), treated with RNAlater and stored at 4°C or −80°C (Methods 3 and 4), and treated with Milk Preservation Solution at room temperature (Methods 5 and 6). Methods 1‐5 were extracted using PowerFood^TM^ Microbial DNA Isolation kit (Mobio), and Method 6 was extracted using Milk DNA Preservation and Isolation kit (Norgen BioTek). At genus level, the most abundant genera were shared across Methods 1‐5. Samples frozen at −80°C had fewest significant changes while samples treated and extracted using Milk Preservation and Isolation kit had the most significant changes when compared to fresh samples. Diversity analysis indicated that variation in microbiota composition was related to the method and extraction kit used. This study highlighted that, when extraction from fresh milk samples is not an option, freezing at −80°C is the next best option to preserve the integrity of the milk microbiome. Furthermore, our results demonstrate that choice of extraction kit had a profound impact on the microbiota populations detected in milk.

## Graphical abstract

The aim of this study was to investigate how different storage methods, temperatures, preservatives, and extraction kits influence the human milk microbiome, compared to fresh samples. This study highlights that when extraction from fresh milk samples is not an option, freezing at −80°C is the next best option to preserve the integrity of the milk microbiome. Furthermore, our results demonstrate that choice of extraction kit had a profound impact on the microbiota populations detected in milk.

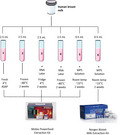

## 1 INTRODUCTION

Human milk was initially considered sterile; however, numerous investigations have since identified milk as an integral source of bacteria for the developing infant (Cabrera‐Rubio et al., 2012, 2019; Hunt et al., 2011; Murphy et al., 2017). Historically, microbiological studies focused on milk contamination due to incorrect collection and storage and subsequent health implications to newborns (Larson et al., 1984; Ryder et al., 1977; West et al., 1979). Culture‐based investigations provided the first evidence of a milk microbiome, although this approach was not without its limitations as some bacterial species are not readily cultivable. Advances in next generation sequencing technologies allowed for more detailed insight into the complex and diverse microbial composition of human milk. This has led to many studies seeking to characterize the milk microbiota, resulting in the identification of over several hundred bacterial species (Le Doare et al., 2018; Lyons et al., 2020).

Variations in the microbial composition of human milk are apparent across many studies. Murphy *et al* reported that the milk microbiome was composed of 12 core genera: *Pseudomonas*, *Staphylococcus*, *Streptococcus*, *Elizabethkingia*, *Variovorax*, *Bifidobacterium*, *Flavobacterium*, *Lactobacillus*, *Stenotrophomonas*, *Brevundimonas*, *Enterobacter*, and *Chryseobacterium*, whereas an investigation by Chen *et al* determined that the 5 core genera predominant in breast milk were *Staphylococcus*, *Streptococcus*, *Enhydrobacter*, *Enterococcus*, and *Rothia* (Chen et al., 2018; Murphy et al., 2017). Additionally, Jost *et al* identified a core microbiota composed of 12 genera: *Staphylococcus*, *Streptococcus*, *Blautia*, *Bifidobacterium*, *Brevundimonas*, *Corynebacterium*, *Flavobacterium*, *Propionibacterium*, *Pseudomonas*, *Ralstonia*, *Rothia*, and *Burkholderia* (Jost et al., 2013). These variations in the core genera of human milk may be attributed to many factors such as maternal health, diet and genetics, mode of delivery, and demographic and environmental differences (Browne et al., 2019; Cabrera‐Rubio et al., 2012; Hermansson et al., 2019; Moossavi et al., 2019). For example, Li *et al* reported geographical differences in the milk microbiome profiles of women living in mainland China and Taiwan, while Kumar *et al* reported differences in the microbiota composition of milk in women from different geographical locations across Europe, Africa, and China (Kumar et al., 2016; Li et al., 2017). More recently, Lackey *et al* documented differences in the microbiota profiles of milk collected from 11 global communities while using standardized extraction methods (Lackey et al., 2019).

However, aside from the influence of maternal and geographical determinants, characterization of the human milk microbiome is subject to many external factors and challenges such as sample collection, storage, and processing which is common with any low biomass sample. Standardization across sample collection, suitable storage conditions, and extraction techniques are essential in order to minimize the risk of contamination from exogenous sources and maintain the integrity of the bacterial community structure in milk (Lackey *et al*., 2019). Ojo‐Okunola *et al* (2020) recently reported substantial differences in the human milk microbiota based on DNA extraction, emphasizing the need for careful consideration when selecting extraction kits. While it is known that cold storage immediately following sample collection and timely DNA extraction is optimum, in certain circumstances these options are not possible. It has been reported that short term cold storage (refrigeration, 4°C) or longer term freezing (−80°C) are the best methods to preserve microbial communities in many biological samples, with a study by Fouhy *et al* reporting that there were no significant differences in fecal microbiota when comparing fresh and frozen samples (Fouhy et al., 2015). Moreover, Hill *et al* documented the success of room temperature transport vials for preserving high diversity microbiota stool samples; however, this storage method is less suitable for low diversity samples such as infant stool samples (Hill et al., 2016). Similarly, Chen *et al* noted that in addition to microbial differences arising from preservation solutions, the 16S rRNA gene primer pair chosen are critical determinants affecting gut microbiota composition (Chen et al., 2019).

To the best of our knowledge, limited research has been performed outlining the microbial differences between fresh milk and frozen milk samples (stored at −80°C) via culture‐independent approaches. Furthermore, if the option of freezing is not available, the addition of preservatives to preserve the bacterial DNA profile in milk may be an alternative. The use of such preservatives to protect the microbial communities in other biological samples has been investigated with differing levels of success (Choo et al., 2015; Hill et al., 2016; Tap et al., 2019). Thus, knowledge on the impact of different storage and extraction methods are needed to assist planning for future projects. Therefore, the aim of this study is to investigate how different storage methods, temperatures, preservatives, and extraction kits influence the overall human milk microbiome, compared to fresh samples using MiSeq sequencing.

## 2 MATERIALS AND METHODS

### 2.1 Participants and sample collection

Eight lactating women were recruited at Cork University Maternity Hospital, Cork, Ireland. All participants were healthy women between 1 and 6 months postpartum. Approximately 15 ml of fresh breast milk was collected from each donor and stored at 4°C until delivery to the laboratory.

### 2.2 Experimental design

Fresh milk samples were treated with six different storage conditions and extraction techniques. One aliquot from each sample was extracted as soon as possible, termed fresh samples (Method 1). The frozen samples were stored at −80°C for two weeks before DNA extraction (Method 2). Two aliquots were subject to treatment with preservative RNAlater (Sigma Aldrich, Arklow, Co. Wicklow, Ireland) in a 2:1 ratio (5 ml RNAlater:2.5 mL milk) followed by storage at either 4 or −80°C for two weeks (Method 3 and Method 4, respectively). The remaining aliquots were treated with a 1:1 ratio of Milk Preservation Solution (Norgen BioTek Corporation, Thorold, Ontario, Canada) (0.5 ml MPS:0.5 ml milk) and stored at room temperature for 2 weeks prior to DNA extraction (Method 5 and Method 6, respectively).

### 2.3 DNA extractions

For Methods 1–4, 2.5 ml of milk was used for extractions and 0.5 ml used for Methods 5 and 6. Microbial DNA was extracted from Methods 1–5 milk samples using a modified protocol from the PowerFood^TM^ Microbial DNA Isolation kit (MoBio, Carlsbad, CA). Briefly, samples were subject to an initial centrifugation 4000 g x 30 min at 4°C, the fat layer was removed with a sterile cotton swab (Thermo Fisher Scientific, Inc.) and supernatant discarded. Cell pellets were washed with phosphate‐buffered saline (Sigma Aldrich) and centrifuged at 13,000 g × 1 min at room temperature. A second wash step was performed using the same process. Samples were treated with 90 µl of 50 mg/ml lysozyme (Sigma Aldrich) and 50 µl of 5 KU/ml mutanolysin (Sigma Aldrich) followed by incubation at 55°C × 15 min. Samples were subsequently treated with 28 µl of 20 mg/ml proteinase k (Qiagen, UK) and incubated further at 55°C × 15 min followed by the Mobio PowerFood^TM^ Microbial DNA Isolation kit protocol. Method 6 used the Milk Preservation and Isolation Kit (Norgen BioTek) as per the manufacturer's instructions with the addition of the optional 2‐h lysozyme step. Two negative controls using sterile molecular water (Sigma Aldrich) were extracted as above, one with each kit.

### 2.4 Preparation of DNA for MiSeq sequencing

Using the 16S metagenomic sequencing library protocol (Illumina) the V3‐V4 hypervariable region of the 16S rRNA gene was amplified from 50 DNA extracts. Template DNA was amplified with V3‐V4 region‐specific primers; forward primer 5’ TCGTCGGCAGCGTCAGATGTGTATAAGAGACAGCCTACGGGNGGCWGCAG; and reverse primer 5’ GTCTCGTGGGCTCGGAGATGTGTATAAGAGACAGGACTACHVGGGTATCTAATCC. Each 25 µl PCR reaction contained template DNA, 1 µl forward primer (5 µM), 1 µl reverse primer (5 µM), 12.5 µl 2× KAPA HiFi Hotstart ready mix (Anachem, Dublin, Ireland), and PCR grade water. PCR conditions for amplification consisted of heated lid 110°, initial denaturation at 95°C × 3 min, 25 cycles of 95°C × 30 s, 55°C × 30 s, 72°C × 30 s, followed by 72°C × 5 min and held at 4°C. PCR products were visualized using gel electrophoresis (1× TAE buffer, 1.5% agarose, 100 V) to confirm DNA amplification. Amplicons were cleaned using AMPure XP magnetic bead‐based purification (Labplan, Dublin, Ireland) and subject to a second PCR reaction. Illumina sequencing adapters and dual‐index barcodes (Illumina Nextera XT indexing primers, Illumina, Sweden) were added to the purified DNA (5 µl) to index each of the samples, allowing the library to be pooled for sequencing. Samples were quantified using the Qubit (Bio‐Sciences, Dublin, Ireland), along with the broad range DNA quantification assay kit (BioSciences) and samples were then pooled in an equimolar fashion. The pooled sample was run on the Agilent Bioanalyser for quality analysis prior to sequencing. The sample pool was prepared following Illumina guidelines. Samples were sequenced on the MiSeq sequencing platform in the Teagasc Sequencing facility, using a 2 x 300 cycle V3 kit, following standard Illumina sequencing protocols.

### 2.5 Bioinformatic and statistical analysis

Three hundred base pair paired‐end reads were assembled using FLASH (FLASH: fast length adjustment of short reads to improve genome assemblies) (Magoč & Salzberg, 2011). QIIME was used for further processing of paired‐end reads, and quality filtering was based on a quality score of >25 and removal of mismatched barcodes and sequences below length thresholds (Caporaso et al., 2010). Denoising, chimera detection, and clustering into operational taxonomic units (OTUs) were performed in QIIME using USEARCH v7 (64‐bit) 3 (Edgar, 2010). OTUs were assigned using PyNAST (PyNAST: python nearest alignment space termination; a flexible tool for aligning sequences to a template alignment) and taxonomic rank was assigned using BLAST against the SILVA SSU Ref database release v123 (Caporaso et al., 2010; Quast et al., 2012). Samples with <15,000 reads were excluded.

Statistical analysis was performed using R (version 3.6.3) and Calypso online software (version 8.84) (Team & R.C., 2013; Zakrzewski et al., 2017). To determine if statistically significant differences occurred in the microbial composition between fresh and each method tested, non‐parametric Mann–Whitney analysis was completed using compareGroups package in R (Avramopoulos, 2017; Subirana et al., 2014). Statistical significance was accepted as *p* < 0.05. In Calypso, cumulative‐sum scaling was used to normalize microbial community data and data were log2 transformed to account for the non‐normal distribution of taxonomic count data for alpha and beta diversity testing. Alpha diversity was determined using the Shannon, Simpson's diversity, and Chao1 indices. Beta diversity was measured using principal coordinate analysis (PCoA) and adonis variance analysis based on Bray–Curtis distance matrices on data. Multivariate analysis was examined using redundancy analysis (RDA) and canonical correspondence analysis (CCA) method to investigate the associations between microbiota composition and explanatory variables.

### 2.6 Quantitative polymerase chain reaction (qPCR)

Absolute quantification by qPCR was used to determine total bacterial numbers in milk samples extracted across different methods using the Roche LightCycler 480 II platform. A standard curve was created using 10^9^ to 10^2^ copies of 16S rRNA/µl to quantify total 16S bacterial counts. Amplification of samples was achieved using the forward primer 5’‐ACTCCTACGGGAGGCAGCAG‐3’ and reverse primer 5’‐ATTACCGCGGCTGCTGG‐3’ and KAPA Lightcycler 480 mix qPCR mix (Anachem, Dublin, Ireland) qPCR conditions were as follows; pre‐incubation at 95°C for 3 min, amplification consisting of 40 cycles at 95°C for 10 s, 60°C for 20 s, 72°C for 1 s, melting curve at 95°C for 5 s, 65°C for 1 min, 97°C continuously and a final cooling at 40°C for 10 s. All samples, negative controls, and standards were run in duplicate.

## 3 RESULTS

**FIGURE 1:**
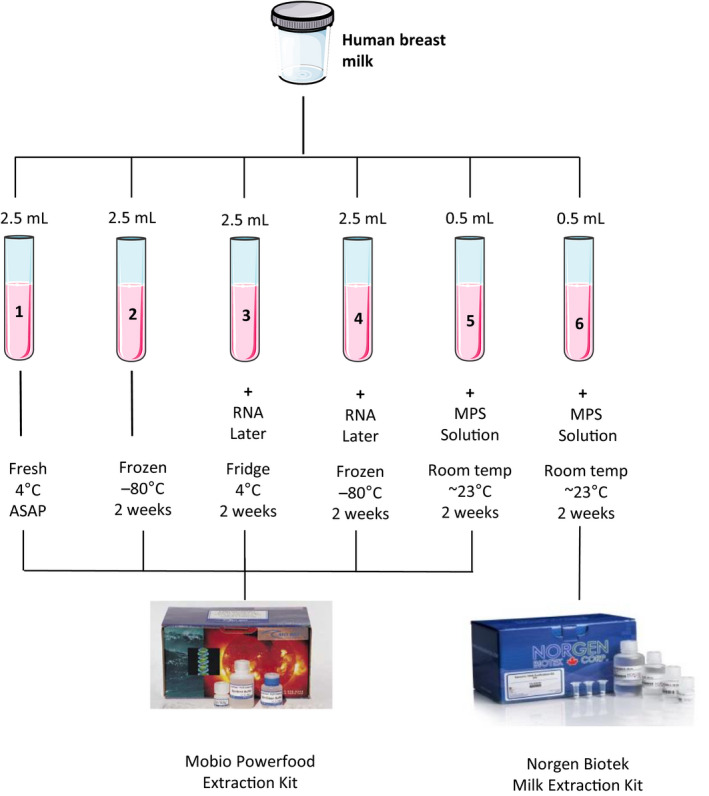
Flow diagram displaying the storage, temperature, and extraction kit to which samples were subjected

In this study, DNA was extracted from human milk samples subjected to six different storage, temperature, and extraction methods (Figure 1). MiSeq sequencing was used to determine the effect of these variables on the milk microbiota when compared to samples extracted from fresh milk.

### 3.1 MiSeq analysis of milk microbiota diversity following different storage and extraction methods

**FIGURE 2:**
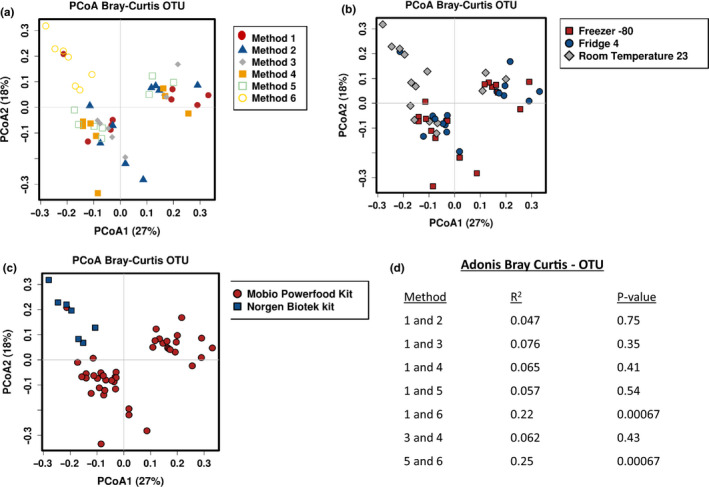
PCoA based on Bray–Curtis operational taxonomic unit (OTU) data on effect of storage and extraction method. Separation of samples based on: a method, b temperature, c extraction kit, and d Adonis variance analysis based on Bray–Curtis distance matrices at OTU

**FIGURE A1:**
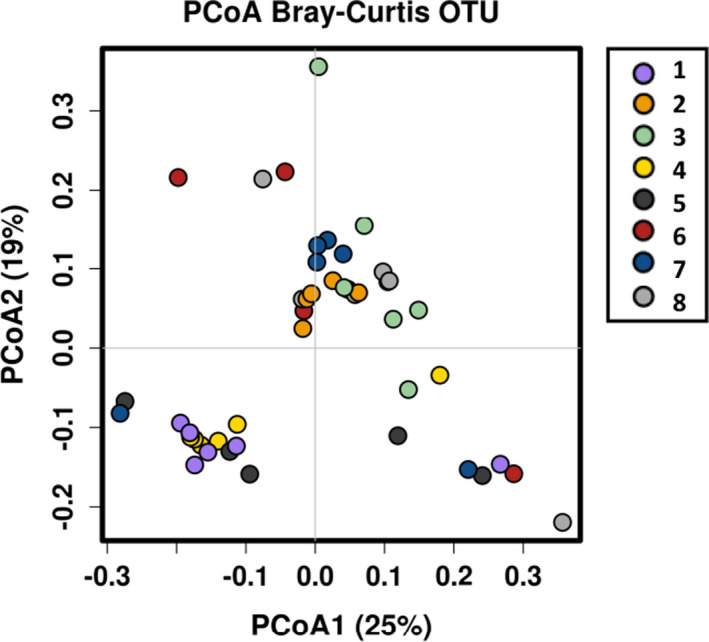
PCoA displaying separation of samples based on mother

To examine the impact of storage conditions and extraction methods on beta diversity, PCoA plots were constructed based on the Bray–Curtis distance matrices at OTU level using relative abundance data. No clear separation based on storage and extraction methods were observed for Methods 1–5; however, milk samples stored and extracted using Method 6 appeared to cluster closely together in the same direction (Figure 2a). There were no obvious clusters of milk samples based on storage temperatures, although separation is apparent in milk samples extracted using different extraction kits (Figure 2b,c). Samples clustered more closely according to the individual, rather than to other samples in the same storage and extraction group except for Method 6 (Appendix Figure A1).

When all methods were compared to samples extracted from fresh (Method 1), the only significant difference was evident between Method 1 and Method 6 (adonis *p* = 0.00067) (Figure 2d). Although Methods 3 and 4 used the same preservative, RNAlater, but were stored at different temperatures (4°C and −80°C), no significant effect was seen between these groups (adonis *p* = 0.43). Despite Methods 5 and 6 using the same milk preservation solution and storage temperature, Method 5 was extracted with the Mobio PowerFood^TM^ Microbial DNA Isolation kit and Method 6 with the Norgen BioTek Milk Preservation and Isolation kit and a significant difference was observed between these groups (adonis *p* = 0.00067).

**FIGURE 3:**
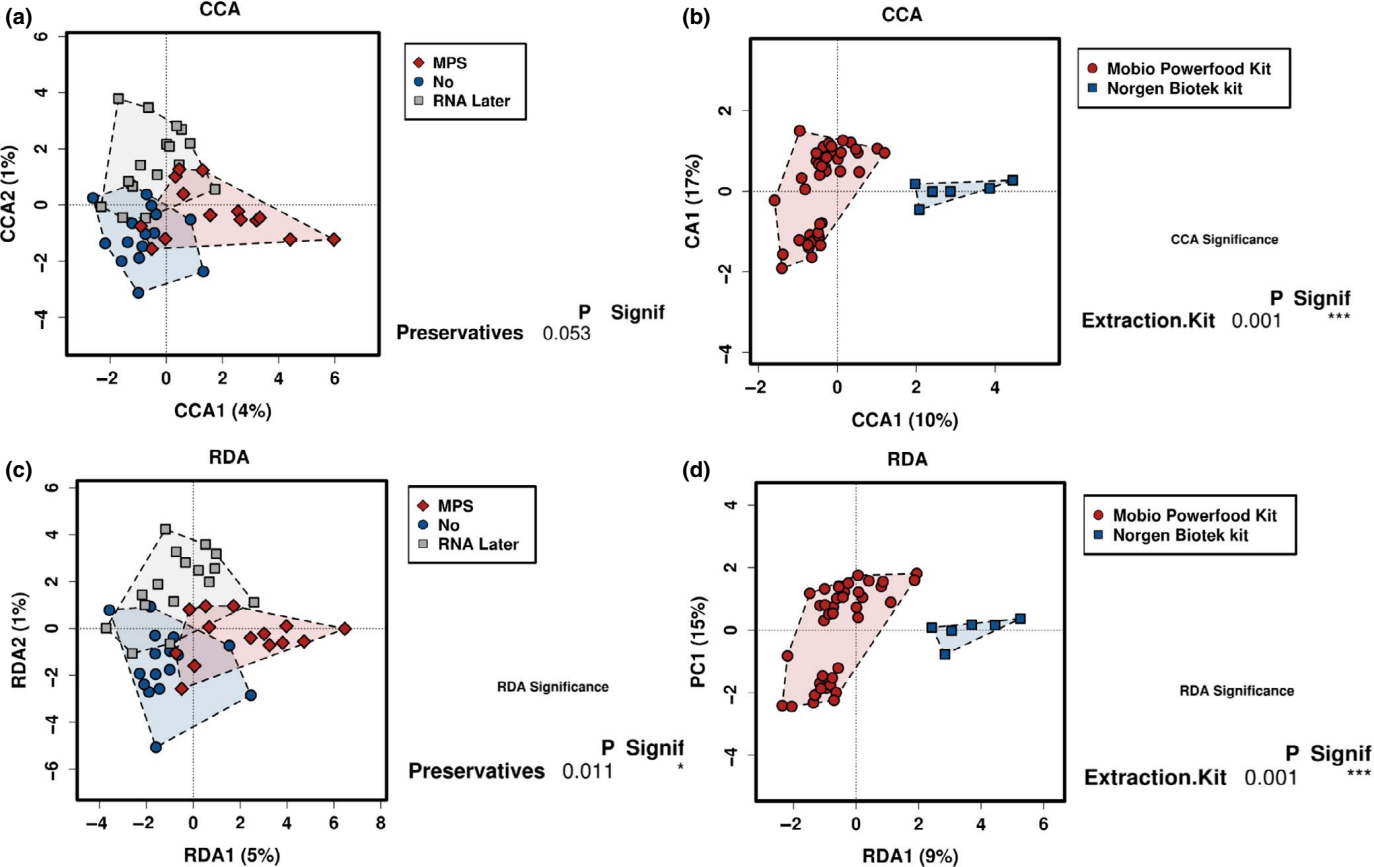
Samples separate based on preservative and extraction kit. a Canonical correspondence analysis (CCA) of OTUs, with samples separating based on preservative and extraction kit. b Redundancy analysis (RDA) of OTUs with samples separating based on preservative and extraction kit

To explore associations between composition and explanatory variables, redundancy analysis plots (RDA) were used and determined that preservative and extraction kit (*p* = 0.01, *p* = 0.001, respectively) had significant impacts on the milk microbiota. In addition, canonical correspondence analysis also determined that extraction kits (*p* = 0.001) had a significant impact on the milk microbiota, while preservatives did not (*p* = 0.053) (Figure 3a,b).

**FIGURE 4:**
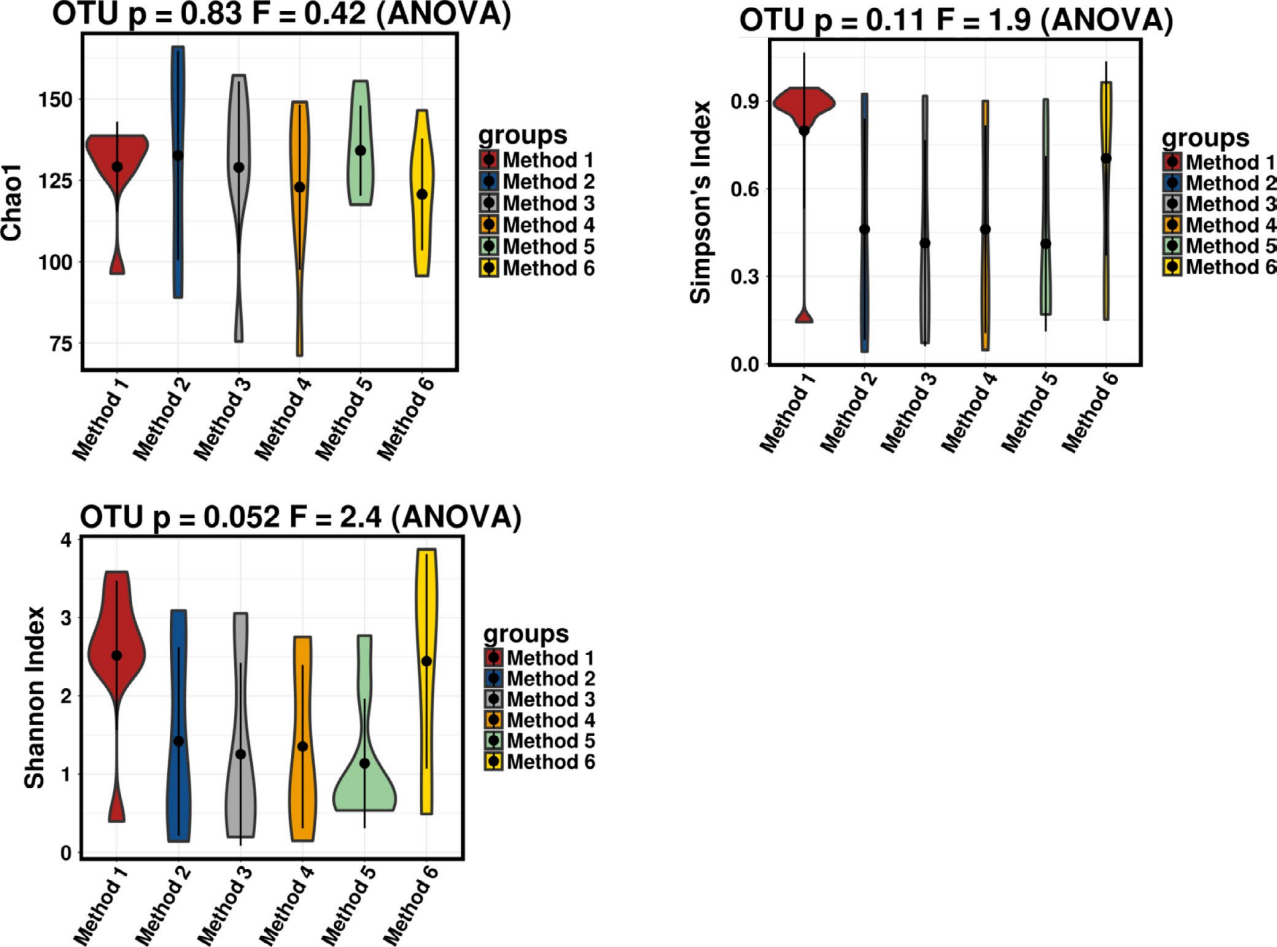
Alpha diversity of samples based on method. No significant differences observed across methods when using Chao1 (*p* = 0.83), Simpson's index (*p* = 0.11), and Shannon index (*p* = 0.052)

To determine if alterations in microbial diversity within samples occurred as a result of different storage and extraction methods alpha diversity was investigated. To estimate microbial richness we used the Chao1 test which showed no significant differences in bacterial richness across the methods (*p* = 0.83). To estimate microbial diversity, we applied Simpson's diversity index, and to predict microbial evenness, we used the Shannon index and both revealed no significant differences across methods (*p* = 0.11 and *p* = 0.052) (Figure 4).

### 3.2 MiSeq analysis of milk microbial composition following different storage and extraction methods

**FIGURE 5:**
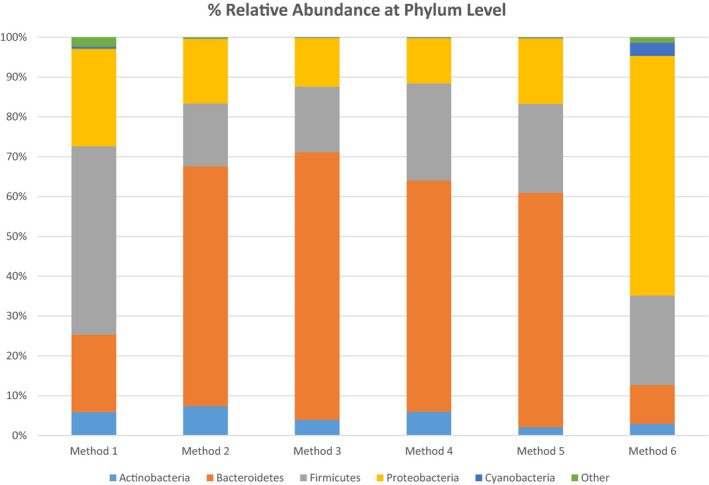
Relative abundances of bacterial phyla in methods 1, 2, 3, 4, 5, and 6. Other contains phyla present at <1% of assignable sequences at phylum level

With regard to phylum, all methods shared common phyla. The predominant phyla across methods were Proteobacteria, Firmicutes, Bacteroidetes, and Actinobacteria, with Cyanobacteria present in higher levels in Method 6 (3.3%) compared with all other methods (<1%). Firmicutes dominated in Method 1 (47%), Bacteroidetes in Methods 2‐5 (58‐67%) and Proteobacteria in Method 6 (60%) (Figure 5) (Table 1). To examine the impact of different methods on the human milk microbiota relative to fresh samples, each method was compared individually against fresh (Method 1) to determine significant changes. Methods 2 and 3 had significantly higher relative abundances of Bacteroidetes (*p* = 0.046, *p* = 0.049, respectively) when compared with Method 1, while Firmicutes were significantly lower in Methods 2, 3, and 6 (*p* = 0.01, *p* = 0.02, *p* = 0.037, respectively). No significant differences were detected in the most abundant phyla between Methods 1 and 4. Relative abundances of Proteobacteria were significantly higher (*p* = 0.01) in Method 6 compared to Method 1.

**TABLE 1:** Mean % relative abundance and standard error of means across methods at phylum level

	Method 1	Method 2	Method 3	Method 4	Method 5	Method 6
Actinobacteria	5.9 ± 1.97	7.39 ± 3.79	3.92 ± 2.36	5.99 ± 2.81	2.08 ± 1.21	2.9 ± 0.94
Bacteroidetes	19.39 ± 11.2	60.2 ± 15.22	67.2 ± 15.54	58.02 ± 16.05	58.92 ± 15.96	9.81 ± 3.07
Firmicutes	47.32 ± 8.55	15.79 ± 4.82	16.46 ± 6.71	24.4 ± 8.74	22.26 ± 9.55	22.46 ± 4.60
Proteobacteria	24.5 ± 7.00	16.27 ± 8.03	12.22 ± 7.74	11.35 ± 5.16	16.48 ± 7.87	60.15 ± 9.15
Cyanobacteria	0.52 ± 0.23	0.17 ± 0.05	0.12 ± 0.03	0.18 ± 0.05	0.15 ± 0.04	3.3 ± 1.15

**FIGURE 6:**
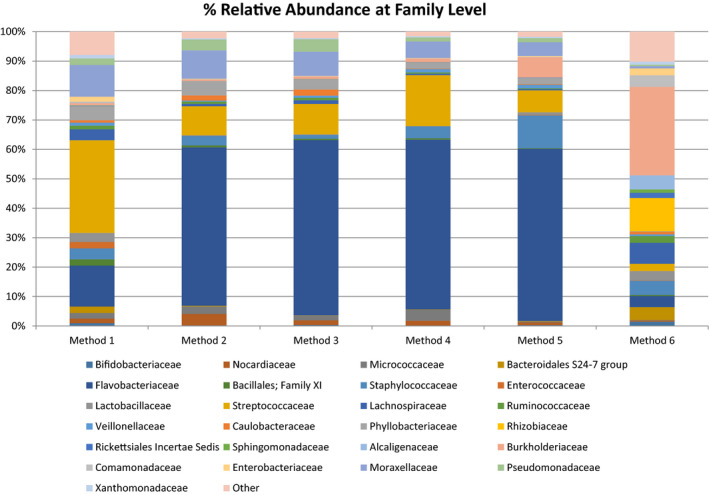
Relative abundances of bacterial families in methods 1, 2, 3, 4, 5, and 6. Other contains families present at <1% of assignable sequences at Family level

The most prevalent families detected in Method 1 (fresh) were *Flavobacteriaceae*, *Streptococcaceae*, *and Moraxellaceae* and were shared across Methods 2‐5. By contrast, the most abundant families in Method 6 were *Burkholderiaceae*, *Rhizobiaceae*, and *Lachnospiraceae*, with *Rhizobiaceae* present in significantly higher levels when compared with Method 1 (Fresh) (*p* = 0.005) (Figure 6).

**FIGURE 7:**
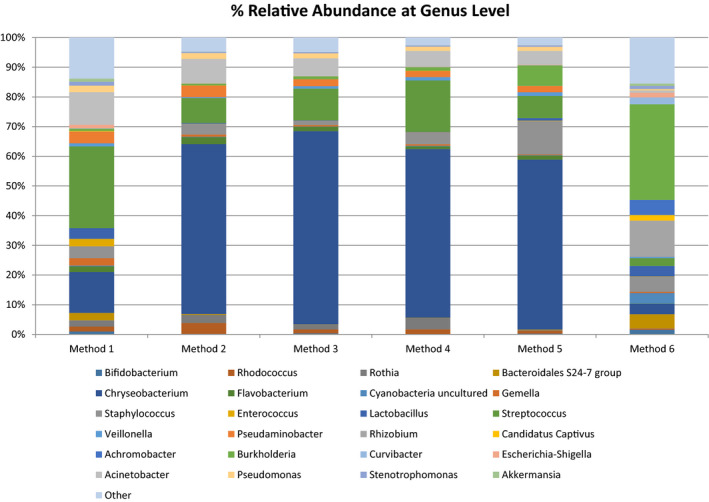
Relative abundances of bacterial genera in Methods 1, 2, 3, 4, 5, and 6. Other contains genera present at <1% of assignable sequences at Genus level

At genus level, the six most abundant genera which appeared to predominate in Methods 1‐5 with a mean relative abundance of ≥1% were *Chryseobacterium*, *Staphylococcus*, *Streptococcus*, *Pseudaminobacter*, *Acinetobacter*, and *Pseudomonas*. These accounted for 60% relative abundance in Method 1, 83% in Method 2, 87% in Method 3, 87% in Method 4, and 84% in Method 5. However, these genera only accounted for 11% of the overall relative abundance in Method 6 where *Burkholderia*, *Achromobacter*, *Rhizobium*, *Staphylococcus*, and an uncultured genus of Bacteroidales S24‐7 were among the most abundant genera with a mean relative abundance of ≥1% (Figure 7) (Table 2). *Streptococcus* was significantly lower in Methods 2 and 6 (*p* = 0.031, *p* = 0.039, respectively) and *Staphylococcus* was significantly lower in Method 3 (*p* = 0.043) when compared with Method 1. No significant differences were observed in the most abundant genera between Methods 1 and 4 and Methods 1 and 5. Relative abundances of *Achromobacter* (*p* = 0.014) and *Rhizobium* (*p* = 0.005) were significantly higher in Method 6 when compared with Method 1.

**TABLE 2:** Mean % relative abundance and standard error of means across methods at genus level

	Method 1	Method 2	Method 3	Method 4	Method 5	Method 6	
*Bifidobacterium*	0.98 ± 0.44	0.19 ± 0.10	0.34 ± 0.17	0.11 ± 0.03	0.28 ± 0.15	1.43 ± 0.75	
*Rhodococcus*	1.59 ± 0.80	3.68 ± 1.92	1.33 ± 0.86	1.63 ± 0.82	0.92 ± 0.74	0.38 ± 0.26	
Rothia	2.04 ± 1.34	2.69 ± 2.58	1.61 ± 1.58	3.79 ± 1.84	0.26 ± 0.18	0.12 ± 0.06	
Bacteroidales S24‐7 uncultured	2.40 ± 1.57	0.27 ± 0.13	0.10 ± 0.03	0.10 ± 0.03	0.16 ± 0.06	4.42 ± 1.55	
*Chryseobacterium*	13.38 ± 11.78	57.06 ± 6.63	64.97 ± 16.84	56.67 ± 16.62	57.13 ± 16.63	3.31 ± 1.00	
*Flavobacterium*	1.87 ± 1.13	2.33 ± 1.31	1.43 ± 1.10	0.97 ± 0.56	1.39 ± 1.19	0.1 ± 0.09	
Cyanobacteria uncultured	0.27 ± 0.20	0.15 ± 0.05	0.12 ± 0.03	0.17 ± 0.05	0.13 ± 0.04	3.26 ± 1.15	
*Staphylococcus*	3.90 ± 0.96	3.60 ± 1.46	1.50 ± 0.76	4.05 ± 3.00	11.16 ± 7.27	4.76 ± 2.20	
*Enterococcus*	2.45 ± 1.57	0.02 ± 0.01	0.02 ± 0.00	0.04 ± 0.03	0.13 ± 0.07	0.08 ± 0.07	
*Lactobacillus*	3.49 ± 3.15	0.23 ± 0.14	0.10 ± 0.04	0.09 ± 0.02	0.90 ± 0.69	3.28 ± 1.66	
*Streptococcus*	26.74 ± 6.40	8.37 ± 3.00	10.62 ± 5.48	17.27 ± 7.82	7.43 ± 4.89	2.43 ± 0.97	
*Lachnospiraceae* NK4A136 group	1.88 ± 1.21	0.19 ± 0.08	0.12 ± 0.04	0.14 ± 0.04	0.16 ± 0.05	4.19 ± 1.50	
*Veillonella*	1.01 ± 0.58	0.30 ± 0.18	0.81 ± 0.73	1.11 ± 0.63	1.22 ± 1.20	0.47 ± 0.22	
*Pseudaminobacter*	3.81 ± 2.09	3.90 ± 2.04	2.34 ± 1.64	2.08 ± 1.09	2.09 ± 1.65	0.08 ± 0.06	
*Rhizobium*	0.04 ± 0.03	0.00 ± 0.00	0.00 ± 0.00	0.00 ± 0.00	0.00 ± 0.00	11.3 ± 5.47	
*Achromobacter*	0.01 ± 0.01	0.00 ± 0.00	0.01 ± 0.00	0.01 ± 0.00	0.01 ± 0.00	4.78 ± 2.90	
*Burkholderia*	0.72 ± 0.45	0.59 ± 0.39	0.95 ± 0.61	1.16 ± 0.57	6.89 ± 4.59	30.08 ± 15.12	
*Curvibacter*	0.13 ± 0.09	0.06 ± 0.02	0.03 ± 0.01	0.04 ± 0.01	0.07 ± 0.02	2.15 ± 0.92	
*Escherichia‐Shigella*	1.18 ± 0.57	0.07 ± 0.03	0.06 ± 0.02	0.09 ± 0.02	0.19 ± 0.12	1.45 ± 1.07	
*Acinetobacter*	10.64 ± 5.07	8.13 ± 4.09	5.96 ± 4.13	5.40 ± 2.75	4.60 ± 3.57	0.711 ± 0.40	
*Pseudomonas*	2.13 ± 1.07	2.00 ± 0.98	1.66 ± 1.11	1.39 ± 0.67	1.37 ± 1.14	0.46 ± 0.16	
*Stenotrophomonas*	1.27 ± 0.49	0.43 ± 0.16	0.43 ± 0.14	0.42 ± 0.09	0.45 ± 0.12	1.00 ± 0.37	

Although few significant differences were observed among the most abundant genera across methods when compared with Method 1, Method 2 had the fewest significant changes across all genera and was most similar to Method 1 (6 significant differences), while Method 6 had the most significant changes across all genera when compared to fresh samples in Method 1 (30 significant changes) (Table3).

**TABLE 3:** Method Comparisons. Significant changes at genus level between fresh (Method 1) and different storage, temperature, and extraction kits (methods 2, 3, 4, 5, and 6) using the Mann–Whitney U test

	Method Comparisons				
	1 and 2	1 and 3	1 and 4	1 and 5	1 and 6
1	*Sediminibacterium* *p* = 0.029	*Propionibacterium* *p* = 0.011	*Bergeyella* *p* = 0.039	*Actinotignum* *p* = 0.02	*Streptomyces* *p* = 0.024
2	*Bacillus* *p* = 0.007	Uncultured Cyanobacteria *p* = 0.017	Uncultured Cyanobacteria *p* = 0.036	*Propionibacterium* *p* = 0.027	*Barnesiella* *p* = 0.011
3	*Streptococcus* *p* = 0.031	Uncultured Cyanobacteria *p* = 0.015	Uncultured Cyanobacteria *p* = 0.017	*Sediminibacterium* *p* = 0.039	Uncultured Cyanobacteria *p* = 0.024
4	Uncultured *Lachnospiraceae* *p* = 0.029	*Staphylococcus* *p* = 0.043	Uncultured Cyanobacteria *p* = 0.007	Uncultured *Christensenellaceae* *p* = 0.029	Uncultured Cyanobacteria *p* = 0.005
5	*Megasphaera* *p* = 0.033	Uncultured *Ruminococcaceae* *p* = 0.034	Uncultured Clostridiales *p* = 0.039	*Allobaculum* *p* = 0.012	*Streptococcus* *p* = 0.039
6	*Schlegelella* *p* = 0.04	*Allobaculum* *p* = 0.042	*Lachnoanaerobaculum* *p* = 0.039	*Solobacterium* *p* = 0.022	*Anaerococcus* *p* = 0.011
7		*Holdemanella* *p* = 0.032	Uncultured *Lachnospiraceae* *p* = 0.02	*Turicibacter* *p* = 0.018	*Anaerostipes* *p* = 0.004
8		Uncultured Alphaproteobacteria *p* = 0.02	*Bilophila* *p* = 0.02	Uncultured *Methylophilaceae* *p* = 0.048	Uncultured *Lachnospiraceae* *p* = 0.011
9		*Sphingomonas* *p* = 0.033		Uncultured Aeromonas *p* = 0.02	*Peptoclostridium* *p* = 0.013
10					*Oscillibacter* *p* = 0.034
11					*Ruminiclostridium* *p* = 0.024
12					*Ruminiclostridium* *p* = 0.039
13					Uncultured *Ruminococcaceae* *p* = 0.016
14					Uncultured *Ruminococcaceae* *p* = 0.03
15					*Holdemanella* *p* = 0.031
16					*Turicibacter* *p* = 0.007
17					Uncultured Fusobacteria *p* = 0.024
18					*Caulobacter* *p* = 0.003
19					*Rhizobium* *p* = 0.005
20					*Candidatus* *Captivus* *p* = 0.012
21					*Novosphingobium* *p* = 0.004
22					*Achromobacter* *p* = 0.014
23					*Ralstonia* *p* = 0.002
24					*Acidovorax* *p* = 0.036
25					*Aquabacterium* *p* = 0.024
26					*Curvibacter* *p* = 0.036
27					*Schlegelella* *p* = 0.024
28					Uncultured *Methylophilaceae* *p* = 0.003
29					*Desulfovibrio* *p* = 0.03
30					*Psychrobacter* *p* = 0.011

Methods 3 and 4 used the same preservation solution, RNAlater, but milk samples were stored at different temperatures prior to DNA extraction, which had no significant impact across the most abundant genera when compared with each other. Some significant differences were observed in genera present at relative abundances of <1% (*Alloprevotella* *p* = 0.039, Uncultured Cyanobacteria *p* = 0.027, and *Catenibacterium* *p* = 0.037). Methods 5 and 6 were both treated with Milk Preservation Solution (MPS) and were stored at room temperature but different extraction kits were used. Over 50 significant changes were detected at genus level between the two methods. The majority of these differences between the groups were among genera present in abundances of <1%. However, *Rhizobium* and *Achromobacter* present at relative abundances of 11% and 5%, respectively, were significantly higher in Method 6 (*p* = 0.001, *p* = 0.012) (Appendix Table A1).

**FIGURE 8:**
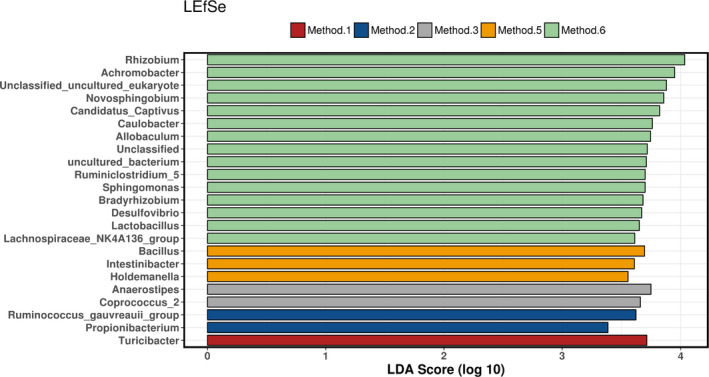
LEfSe analysis determining discriminative taxa across all methods at genus level

To determine whether there were bacteria present in the milk microbiota that could discriminate based on method used, a feature selection statistical analysis LDA effect size (LEfSe) which determines the most likely taxa to explain differences between the groups was carried out at genus level. Method 6 had the highest number of discriminative genera when compared to fresh and all other methods. There were 15 genera that discriminated Method 6, with *Rhizobium* having the greatest discriminatory power for Method 6 compared to all other methods (Figure 8).

After sequencing, filtering, and quality control, the Mobio Powerfood kit and Norgen Biotek kit negative controls yielded extremely low reads of 89 and 3 respectively. Due to low sequence reads, genera identified in the negative controls did not impact the microbiome of samples in this study (Appendix Table A2).

### 3.3 Quantitative polymerase chain reaction (qPCR) of bacterial counts across methods

**FIGURE 9:**
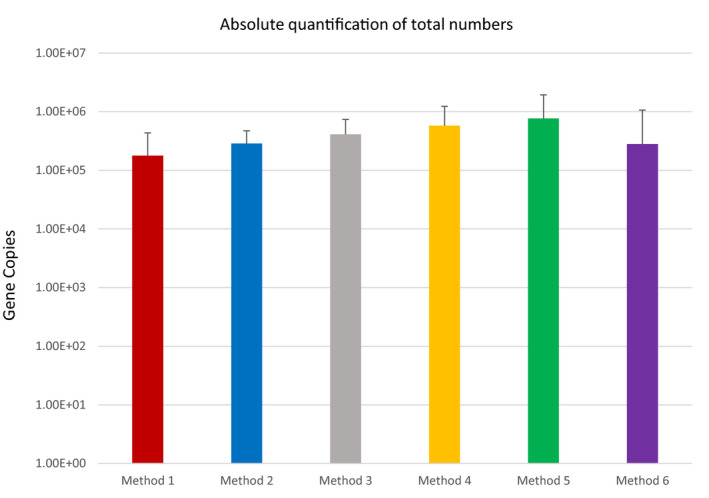
Total bacterial numbers (gene copies) as determined by 16S rRNA qPCR analysis for each method

To determine if storage method and extraction kit had an impact on total bacterial counts in the milk microbiota, total 16S rRNA levels were determined by qPCR. Total gene copies were detected at similar levels across all groups and no significant differences were observed when methods were compared to fresh samples (Method 1) (Figure 9). It was established that storage method and extraction kit did not significantly impact total bacterial numbers.

## 4 DISCUSSION

Advances in next generation sequencing have enabled detailed insight into the complex microbial ecosystems of many biological samples including human milk. However, many factors can influence these findings including sample handling and storage methods, temperatures, and extraction kits (Fouhy et al., 2015; Kennedy et al., 2014). While some previous studies have examined these factors, to the best of our knowledge, this is the first study to examine the effect of freezing at −80°C on the human milk microbiota when compared to fresh samples using MiSeq sequencing. Furthermore, few studies have examined the effect of preservatives and extraction kits on the human milk microbiota (Douglas et al., 2020; Lackey et al., 2017). Our aim was to determine if alterations in the milk microbiome occur following freezing and cold storage of milk samples, and if preservation solutions and extraction kits alter the integrity of the milk microbiome. While DNA extraction from fresh samples is regarded as the optimum method, it may not be feasible in many large‐scale studies where high numbers of samples are collected. Furthermore, sample collection from participating mothers may occur at times where immediate extraction is not possible thus furthering the need to determine other storage methods which preserve the bacterial communities in milk. Therefore, it is imperative to understand the effects of different storage conditions and extraction kits on the human milk microbiota for future study designs, sample processing, analysis, and comparisons with other studies.

There were six methods tested in this study to assess the impact on the microbiota of human milk. DNA extraction from freshly collected samples is considered the gold standard method (Cuív et al., 2011; Hill et al., 2016; Maukonen et al., 2012; Wu et al., 2010), however, alternative options also need to be investigated when this is not possible. The extraction kits used in these investigations were the Mobio PowerFood^TM^ Microbial DNA Isolation kit and the Norgen Milk DNA Preservation and Isolation kit. The Mobio PowerFood^TM^ Microbial DNA Isolation kit has been used across multiple studies to extract DNA from cow's milk (Dahlberg et al., 2019; Lima et al., 2018; Quigley et al., 2013), and was determined to be most consistent for the extraction of highly concentrated and pure DNA for subsequent use in downstream sequencing applications when compared with the other extraction kits (Quigley et al., 2012). The Milk DNA Preservation and Isolation kit is designed specifically for use with milk samples.

The preservatives used in this study were selected after reviewing the literature on different solutions used to maintain the microbial composition of biological samples. RNAlater is a tissue storage reagent designed to stabilize and protect RNA. Although studies have investigated its ability to preserve DNA in fecal samples (Al et al., 2018; Flores et al., 2015; Hickl et al., 2019; Liang et al., 2020; Tap et al., 2019) and human milk samples when stored at 37°C (Lackey et al., 2017), no studies have looked at the ability of RNAlater to preserve and stabilize the bacterial communities in milk when stored at 4°C or frozen at −80°C (Methods 3 and 4, respectively)., It has been reported that treatment with MPS accompanying the Milk DNA Preservation and Isolation kit can preserve the bacterial integrity of milk when stored at room temperature and at 37°C (Lackey et al., 2017). In addition to preserving the bacterial communities in milk by preventing the growth of Gram‐negative and Gram‐positive bacteria, a significantly lower volume of milk is needed for extraction with this kit when compared to other extraction kits. For Method 5, this MPS was also used in conjunction with the Mobio PowerFood^TM^ Microbial DNA Isolation kit, which was used across Methods 1–5 to determine if kit choice impacted the microbiota profile. Our analysis revealed that samples preserved and extracted using the Milk DNA Preservation and Isolation kit appeared to have the most bacteriologically different compositions when compared with fresh samples.

16S rRNA compositional sequencing was used to determine the impact of different processing methods on the microbiota. Our data show that the microbiota of fresh samples were most similar to samples frozen at −80°C for two weeks (Method 2) when compared to all methods. Although a significant difference in *Streptococcus* was observed between these methods, overall, frozen samples had the fewest significant differences when compared with fresh samples. While Method 2 had the fewest significant changes, Methods 3, 4, and 5 also displayed minimal significant differences to fresh samples. Method 3 in which milk samples were preserved with RNAlater and stored at 4°C for two weeks showed significantly lower *Staphylococcus*; however, this finding was not observed in Method 4 which was also treated with RNAlater but frozen at −80°C for two weeks. Furthermore, Method 4 had less overall significant differences compared to fresh samples than Method 3, indicating that freezer storage at −80°C appears to be better at preserving the microbiota of milk than cold storage at 4°C for the same amount of time. Thus far, our results demonstrate that when processing of fresh samples is not feasible, storage at −80°C gives the most accurate microbiota representation. This finding coincides with a previous study which found minimal significant differences between the microbiota of fresh, frozen and snap frozen fecal samples and determined that frozen and snap frozen samples gave accurate microbiota profiles (Fouhy et al., 2015).

Our data show that samples treated with MPS and extracted using the Milk Preservation and Isolation kit (Method 6) were most different in bacterial composition to fresh samples. Common taxa were shared across Methods 1–5; however, at family and genus level the most abundant families and genera differed in Method 6 when compared with the other groups. Method 6 displayed the greatest differences at family and genus level compared with fresh samples. Taking into account that Method 5 was treated with the same MPS as Method 6, the only variable between these methods is the extraction kit, which appears to account for the differences when compared with Method 1. When Methods 5 and 6 were compared with each other, over 50 significant differences among genera were found. Thus, our results appear to demonstrate that extraction kits can affect bacterial communities in milk. A similar finding was observed by Douglas *et al* who noted that different extraction kits and methodologies resulted in differing 16S microbiota profiles of milk samples (Kennedy et al., 2014). Furthermore, Method 6 resulted in the detection of taxa not identified in Method 1. When compared with Method 1, *Streptococcus* appeared to be underrepresented in Method 6 falling from 31% to 2%. Lackey et al. (2017) also reported that samples preserved and extracted using the Milk Preservation and Isolation kit had lowest relative abundance of *Streptococcus* when compared with other samples. As the Gram‐negative Proteobacteria were significantly higher in Method 6, we hypothesize that some preservatives and extraction kits are potentially better at lysing and extracting according to Gram status resulting in a biased microbiota profile. Our study determined that MPS in conjunction with the Milk Isolation kit may favor the isolation of DNA from Gram‐negative bacteria. It has also been reported that different collection methods influenced the microbiota of fecal samples, with the Stool Nucleic Acid Collection and Preservation tubes (Norgen Biotek) resulting in the detection of increased Gram‐negative bacteria (Watson et al., 2019). Moreover, with regard to extraction kits chosen, the different nature of the protocols could explain the differing bacterial composition. It has been documented that the method of cell lysis can significantly influence the microbial communities, with methods using mechanical lysis resulting in higher amounts of DNA at a higher quality. The Mobio Powerfood kit incorporates both mechanical (bead‐beating) and enzymatic lysis steps whereas the Norgen Biotek kit employs solely enzymatic lysis steps. It is also worth noting the importance of including negative controls in extractions and sequencing in order to ensure accuracy of results and eliminate any potential contaminants overriding the microbiome especially with low biomass samples. While it has been reported that some taxa are commonly identified in the “kitome” (Olomu et al., 2020; Salter et al., 2014; Weyrich et al., 2019), the low sequencing reads obtained in this study indicate the reagents were not contaminated and did not impact the taxa observed across samples in this study. Furthermore, in addition to extraction kit, multiple negative controls should be included throughout sequencing preparation in order to account for spurious sequences and identify contamination that may occur as reported previously (Hornung et al., 2019; Kim et al., 2017). Failure to identify contamination may lead to unreliable and inaccurate data and results, which is a pitfall of many low biomass studies.

This study also examined microbial diversity and found no significant differences between the methods in terms of alpha diversity. However, Adonis variance analysis based on Bray–Curtis distance matrices found significance between Methods 1 and 6, and Methods 5 and 6, suggesting that the choice of extraction kit may be driving the separation between samples.

Although our study looked at short term storage (2 weeks) using different temperatures and extraction methods, further investigations are necessary to determine if prolonged storage has any significant effect on the milk microbiome composition. While processing samples from fresh is regarded as optimal, and we have used it as the method of comparison in our study, it was not known what the true microbiome composition of the milk samples were prior to processing. We are aware of the limitations of this study, and future research would benefit from the addition of a mock microbial community in order to determine the exact composition, and subsequent effect of storage temperature and extraction methods on the microbiota populations detected in milk.

## 5 CONCLUSIONS

In conclusion, if processing human milk samples from fresh is not feasible, other storage conditions are needed to preserve the microbial integrity of milk. When considering the methods used in this study, samples frozen at −80°C revealed a microbiota profile closest to that of fresh samples. Samples preserved using MPS and extracted using Milk Preservation and Isolation kit (Method 6) resulted in a significantly different microbiota than that of fresh samples. Our results suggest that freezing samples at −80°C is the most suitable storage method of milk samples prior to extraction when processing fresh samples is not feasible. This knowledge is of vital importance when planning future large‐scale projects, and it will be essential to consider how samples were stored and processed when comparing data from different studies.

## CONFLICT OF INTEREST

None declared.

## ETHICS STATEMENT

This study was approved by the Clinical Research Ethics Committee of the Cork Teaching Hospitals, Cork, Ireland (ethical approval reference: ECM (rr) 21/03/17). Participants provided written informed consent, and all relevant guidelines and regulations were followed.

## AUTHOR CONTRIBUTION

**Katriona E Lyons:** Data curation (equal); Formal analysis (lead); Investigation (lead); Methodology (lead); Writing‐original draft (lead); Writing‐review & editing (equal). **Fiona Fouhy:** Data curation (equal); Methodology (equal). **Carol‐Anne O'Shea:** Resources (lead). **Anthony Ryan:** Conceptualization (equal); Writing‐review & editing (equal). **Eugene Dempsey:** Conceptualization (equal); Resources (equal). **Paul Ross:** Conceptualization (lead); Supervision (lead); Writing‐review & editing (lead). **Catherine Stanton:** Conceptualization (lead); Funding acquisition (lead); Supervision (lead); Writing‐review & editing (lead).

## Data Availability

All data provided in the results section of this study are supported by the Source Data file available in figshare at https://doi.org/10.6084/m9.figshare.12951002. The Source Data file includes the raw sequencing data, table_tax file (Calypso V3 format) and metadata required for each figure in the statistical analysis in this study.

## Appendix

**TABLE A1:** Method comparisons displaying significant genera between methods 3 and 4, and methods 5 and 6

		*p* Value	Method comparisons	*p* Value
	3 and 4		5 and 6	
1	*Alloprevotella*	0.039	*Methanobrevibacter*	0.038
2	Uncultured Cyanobacteria	0.027	*Actinomyces*	0.038
3	*Catenibacterium*	0.037	*Actinotignum*	0.038
4			*Corynebacterium*	0.038
5			*Rhodoglobus*	0.033
6			*Streptomyces*	0.016
7			*Bacteroidales S24.7 group*	0.008
8			*Barnesiella*	0.038
9			*Parabacteroides*	0.038
10			*Spirosoma*	0.047
11			*Sediminibacterium*	0.017
12			*Boechera gunnisoniana*	0.006
13			Cyanobacteria uncultured	0.015
14			*Kurthia*	0.016
15			*Anaerococcus*	0.016
16			*Anaerostipes*	0.002
17			*Dorea*	0.038
18			*Lachnospiraceae NC2004 group*	0.016
19			*Lachnospiraceae NK3A20 group*	0.016
20			*Lachnospiraceae NK4A136 group*	0.037
21			*Lachnospiraceae UCG.004*	0.038
22			*Eubacterium ventriosum*	0.038
23			*Lachnospiraceae uncultured*	0.037
24			*Intestinibacter*	0.018
25			*Peptoclostridium*	0.01
26			*Oscillibacter*	0.012
27			*Ruminiclostridium*	0.038
28			*Ruminiclostridium 5*	0.021
29			*Allobaculum*	0.025
30			*Holdemanella*	0.017
31			*Solobacterium*	0.002
32			*Brevundimonas*	0.021
33			*Caulobacter*	0.005
34			*Rhizobium*	0.001
35			*Candidatus Captivus*	0.037
36			*Novosphingobium*	0.008
37			*Achromobacter*	0.012
38			*Sutterella*	0.038
39			*Ralstonia*	0.002
40			*Aquabacterium*	0.038
41			*Pelomonas*	0.049
42			*Schlegelella*	0.038
43			*Hydrogenophilus*	0.016
44			*Methylophilaceae OM43*	0.032
45			*Desulfovibrio*	0.005
46			Uncultured *Aeromonas*	0.038
47			*Rheinheimera*	0.017
48			*Rahnella*	0.032
49			*Halomonas*	0.038
50			*Haemophilus*	0.038
51			*Psychrobacter*	0.006
52			*Thermomonas*	0.038

**TABLE A2:** Number of assigned sequencing reads and mean relative abundance of genera detected in negative controls

	MoBio PowerFood control	Norgen BioTek control
Number of sequencing reads	89	3
Genera detected (% Relative Abundance)		
*Bifidobacterium*	2.04	
Uncultured Bacteroidales	1.02	
*Alistipes*	4.08	
*Chryseobacterium*	9.18	
*Flavobacterium*	2.04	
Uncultured Cyanobacteria	2.04	33.33
*Staphylococcus*	14.29	
*Streptococcus*	4.08	33.33
*Lachnospiraceae NK4A136 group*	1.02	
*Tyzzerella 4*	1.02	
*Ruminococcaceae NK4A214 group*	1.02	
*Turicibacter*	1.02	
*Fusobacterium*	1.02	
*Brevundimonas*	4.08	
*Caulobacter*	1.02	
*Mesorhizobium*	3.06	
*Pseudaminobacter*	1.02	
*Rhizobium*	21.43	
*Novosphingobium*	1.02	
*Sphingomonas*	1.02	
*Achromobacter*	10.20	
*Burkholderia*	3.06	
*Desulfovibrio*	1.02	
*Escherichia‐Shigella*	2.04	
*Acinetobacter*	2.04	
*Pseudomonas*	4.08	
*Stenotrophomonas*	1.02	
*Rhodococcus*		33.33
